# Endometrial carcinoma located in the right septate uterus cavity: a case report

**DOI:** 10.11604/pamj.2015.22.272.7682

**Published:** 2015-11-20

**Authors:** Ikram Boubess, Youssef Mahdi, Hanan Ramsiss, Adib Filali, Mohamad Hassan Alami, Basma El khannoussi, Hafid Hachi

**Affiliations:** 1 National Centre of Reproductive Health, University of Hospital Ibn Sina, Rabat Morocco; 2 Departement of Pathology, National Institute of Oncology, University Hospital Ibn Sina, Rabat Morocco; 3 Pole of Gynecologic Breast Surgery, National Institute of Oncology, University Hospital Ibn Sina, Rabat Morocco

**Keywords:** Endometrial cancer, uterine malformation, septate uterus

## Abstract

Endometrial cancer in patients with uterine congenital malformations is exceptional and there are only a few rare cases published in the literature. We report the case of a 67 years-old patient with an endometrial cancer located in the right cavity of a complete septate uterus.

## Introduction

Congenital uterine malformations result from a total or incomplete caudal migration of Müllerian ducts which is responsible for uterine atresia, hypoplastic or Aplastic uterus or from a fusion failure of ducts responsible for uterine duplication. Failure of resorption of septa between the mullerian ducts leads to the formation of septate uterus. The incidence of these malformations is estimated around 4-5% [1] but the rate seems to be higher since they are usually discovered as part of an infertility evaluation or repeated miscarriages. Endometrial cancer in patients with these abnormalities is rarely described. We report the case of a patient with unrecognized complete septate uterus associated to endometrium cancer located in the right hemicavity.

## Patient and observation

**Figure 1:**
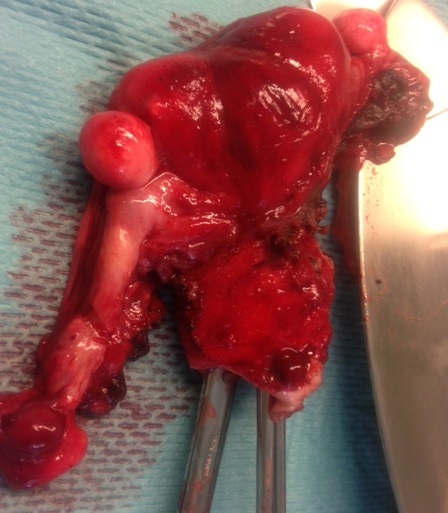
Normal external configuration of the septate uterus

**Figure 2:**
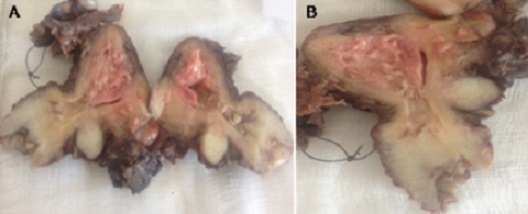
(A) et (B) pathology spicemen shown a complete septate uterus with whitish tumor process in the right hemiuterus and apparently normal left hemiuterus

**Figure 3:**
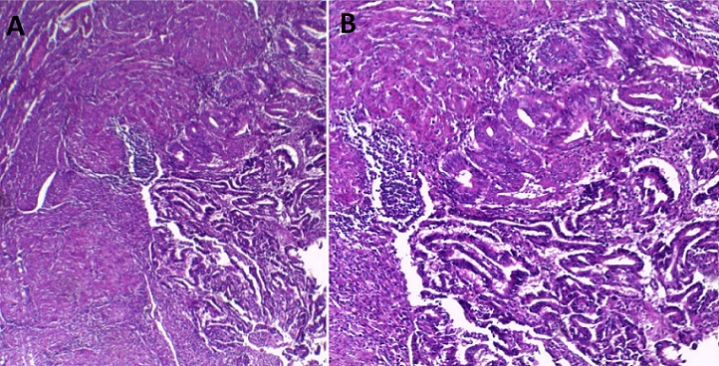
Histological aspect of the tumor (H & E). Crowded and irregularly shaped glands lined by moderately atypical cells, separated by a little stroma (A:×40, B:×100)

A 67- year-old woman with no medical history, nulligravida, post-menopausal since the age of 52, consulted for vaginal bleeding for about 2 weeks. Clinical examination revealed an endocervical bleeding without any suspicious vaginal or cervical lesions. Transvaginal Ultrasound showed an endometrial hypertrophy and an endometrial curettage biopsy performed afterwards revealed a poorly differentiated cancerous process. The patient had an RMI that objectified a complete septate uterus with a 40 mm tumor process limited to the right side. A total abdominal hysterectomy, bilateral-salpingo-oopho-rectomy and staging (bilateral pelvic lymph node dissection and pelvic washings) were performed. Pathological examination of the surgical specimen showed a macroscopically uterus 9x7, 5x2cm with normal external configuration (Figure 1). The coronal examination confirmed the presence of a total septum reaching the cervical canal (Figure 2) with a 4.9 cm long axis whith a whitish tumor process in the right cavity. The microscopic study revealed the presence of a tumor lesion corresponding to an endometrioid adenocarcinoma grade I of the WHO classification that infiltrated more than the one half of the myometrium without any peritumoral vascular embolus (Figure 3). In the left side, an atrophic endometrium was found. The bilateral adnexae, parametia and pelvic lymph nodes were all free of tumor.

## Discussion

There are several classifications to categorize uterine malformations; however, the last anatomical classification seems to be the more adapted one it [2]. In the case of septate uterus, the external configuration is normal because the default of resorption of septum between the Müllerian ducts happens at an advanced stage. The correlation between these abnormalities and the incidence of endometrial cancer has never been established and some authors suggest that Mullerian abnormalities can be a protective factor against endometrial cancer by hormone abnormalities signals to estrogen receptors [3]. The Mullerian abnormalities associated with endometrial cancer are exceptionally described. A few cases of endometrial cancer arising in bicornuate uterus have been reported [4, 5]. Most reports showed endometrial adenocarcinoma involving one horn [6, 7] and only two cases reported the endometrial adenocarcinoma in both horns [5, 8]. After consulting the pubmed database, we found two publications on the location of endometrial adenocarcinoma in one side of a septate uterus. The first reported case was discovered incidentally during a hysteroscopy diagnosis [9]. The second case was discovered in a patient during the exploration of post partum menometrorrhagia [10]. Our case is the first case reported describing an endometrial cancer in a complete septate uterus in a patient with post-menopausal bleeding. The problem in this association is to confirm the malignancy of a suspicious cavity image especially when there is an incorrect appreciation of these defects or in case of focal lesion that may be missed by curettage [11]. Lopez-Fernandez highlights the importance of biopsy by hysteroscopy in case of uterine malformations [9].

## Conclusion

The combination of a uterine malformation to malignant tumor pathology is exceptional and poses the problem of confirmation in some situations in which hysteroscopy is the key element of the management.
